# Psychometric properties of the Swedish childbirth self-efficacy inventory (Swe-CBSEI)

**DOI:** 10.1186/1471-2393-14-1

**Published:** 2014-01-03

**Authors:** Ing-Marie Carlsson, Kristina Ziegert, Eva Nissen

**Affiliations:** 1 Department of Women’s and Children’s health at Karolinska Institutet, SE-171 77 Stockholm, Sweden; 2 School of Social and Health Sciences, Halmstad University, SE-823, SE-301 18 Halmstad, Sweden; 3 Halmstad County Hospital, SE-301 85 Halmstad, Sweden; 4 School of Social and Health Sciences, Örebro University, SE-701 82 Örebro, Sweden

**Keywords:** Childbirth, Self-efficacy, Instrument development, Psychometric properties, Principal component analysis, Think aloud, Midwifery

## Abstract

**Background:**

Previous research has reported that women who are admitted to delivery wards in early labour process before an active stage of labour has started run an increased risk of instrumental deliveries. Therefore, it is essential to focus on factors such as self-efficacy that can enhance a woman’s own ability to cope with the first stage of labour. However, there was no Swedish instrument measuring childbirth self-efficacy available. Thus, the aim of the study was to translate the Childbirth Self-efficacy Inventory and to psychometrically test the Swedish version on first- time mothers within the Swedish culture.

**Methods:**

The method included a forward-backward translation with face and content validity. The psychometric properties were evaluated using a Principal Component Analysis and by using Cronbach’s alpha coefficient and inter-item correlations. Descriptive statistics and non-parametric tests were used to describe and compare the scales. All data were collected from January 2011 to June 2012, from 406 pregnant women during the gestational week 35-42.

**Results:**

The Swedish version of the Childbirth Self-Efficacy Inventory indicated good reliability and the Principal Component Analysis showed a three-component structure. The Wilcoxon Signed-Ranks Test indicated that the women could differentiate between the concepts outcome expectancy and self-efficacy expectatancy and between the two labour stages, active stage and the second stage of labour.

**Conclusions:**

The Swedish version of Childbirth Self-efficacy Inventory is a reliable and valid instrument. The inventory can act as a tool to identify those women who need extra support and to evaluate the efforts of improving women’s self-efficacy during pregnancy.

## Background

Childbirth is a life event that has a lifelong impact on women’s lives [1]. Sometimes the birth process deviates from this otherwise normal event and results in an emergency caesarean section. Today, this area is topical since there is a rising number of caesarean sections in the world, and this also applies to Sweden [2]. This increase has not led to better health outcomes neither for the women nor their children. Instead this complication may have far-reaching negative influence on both women’s physical health [3-6] as well as their mental health [7].

It has been well-documented that one group of women that runs an increased risk of instrumental deliveries are women who are admitted to delivery wards in the early stage of labour before the stage of active labour has started [8-10]. Bailit et al. [8] found that the frequency of emergency caesarian section was twice that of women admitted to delivery wards in early labour compared to women who sought care in the active phase of labour. Moreover, the risk for an abnormal birth outcome in relation to early admission is particularly high if the woman is also expecting her first child [9,11,12]. The reason for this deviation from the normal birthing process is still not clear.

In our research programme about early labour process, where this study is included, we began inductively with two qualitative studies on women’s experiences and coping strategies during the early labour process [13,14]. The results from the first study [13] showed that women who choose to seek care in the early stage of labour had a need “to hand over responsibility for themselves, for the childbirth process and the child’s welfare to the professionals”. These women’s experiences of the latent phase of labour were quite dreadful, with descriptions of a never-ending painful process that could also cause feelings of helplessness [13]. These findings raised further questions. Therefore, in the second study, we explored the experiences of early labour from women who choose to remain at home until the active stage of labour had set in [14]. The findings of this study were diametrically different from the first study. The women in this second study described that they had some kind of power within themselves that was seen as an innate feminine gift, i.e. bodily and mental strength. This power made them feel that they had the ability to cope with the impending birth. One hypothesis raised by these various findings, was that the coping strategies were based on differences in women’s confidence in their ability to give birth, synonymous with Bandura’s self-efficacy theory [15].

### The construct self-efficacy related to childbirth

Bandura’s Social Cognitive Theory [15], with the construct of self-efficacy, describes an individual’s belief in their own ability to behave in a particular way in a specific situation. Self-efficacy is like a cognitive picture, an internal image of how individuals judge themselves being able to execute courses of action required in a particular situation. Self-efficacy consists of cognitive, emotional and behavioural components that are interrelated and affect each other. It means that self-efficacy beliefs are relevant to how people feel, think and motivate themselves, which in turn, affect their choices and behaviour [16,17].

Most women in the western world experience childbirth only a few times. This means that labour and birth is a rather unknown situation for the woman. Facing an unknown situation requires new knowledge and new skills, and may thereby cause uncertainty which is a natural feeling when encountering difficulties and new situations [18]. In this new situation the woman must assess what particular behaviours are required to cope with this situation (i.e. outcome expectancy), and to judge whether she has the ability successfully perform these skills (i.e. self-efficacy). A person’s estimated level of self-efficacy is related to a specific situation, in this case giving birth [16,18].

As mentioned above, self-efficacy beliefs affect the motivation and individuals with high self-efficacy tend to face the situation without resistance, make more effort and persist longer in adversity than individuals with low self-efficacy. In contrast, people who doubt their capabilities tend to avoid situations that they believe exceed their efficacy and have a higher propensity to view the situation as a threat [16,18].

Findings from childbirth research have shown that self-efficacy affect women’s coping and experiences of childbirth [19,20]. Lowe [20], reports that women with low self-efficacy experience more fear of giving birth as well as fear of losing control during delivery. Moreover, these women express more fear for labour pain. The experience of labour pain has in turn been shown to be associated with a woman’s self-efficacy. Women who express greater confidence in their ability to cope with labour also report feeling less pain during labour [21-23]. Self-efficacy is a potent predictor of behaviour [17,24] and therefore of interest in current research. It may be that a woman’s belief in her own ability to handle the imminent birth influences how she is capable of staying home during the early stage of labour. To investigate this hypothesis a valid measurement had to be used and we found Lowe’s instrument Childbirth Self-efficacy Inventory [25].

### The childbirth self-efficacy inventory

The Childbirth Self-Efficacy Inventory (CBSEI) was developed by Nancy K Lowe [25] in USA. It was initially generated through content analysis of post-partum interviews with both nulliparous and multiparous women. The CBSEI is based on Bandura’s self-efficacy theory [18]. When Lowe developed the original CBSEI she found support of construct validity with generalized self-efficacy, self-esteem and internal health locus of control [25]. The CBSEI was in turn negatively correlated with external health locus of control [25].

The inventory is a self-report instrument that includes 62- items. It is divided into four subscales measuring both outcome expectancies, i.e. what behavior they think would be useful during labour as well as self-efficacy expectancies, i.e. how they think they will be able to conduct themselves during labour. The instrument measures these two dimensions of the self-efficacy construct, during both the first and the second stage of the labour process. The responses are distributed by a 15-item scale for the outcome expectancy scales and a 16-item scale for the self-efficacy expectancy scales.

The response rates for all four scales range from 1 to 10; higher scores indicate a higher degree of childbirth self-efficacy.

The instrument is meant to be applied in late pregnancy during the third trimester to estimate the woman’s confidence in childbirth before the impending birth. The CBSEI has been shown to be a psychometrically reliable instrument with high internal consistency measured by Cronbach’s alpha with values above 0.90 on all four subscales [19,25,26]. The inventory has also proved to be a valid tool as reported in multiple studies, using factor analysis as the tool to identify underlying concepts [19,25,27-30]. Moreover, besides the original language English, it has been translated into several other languages: Chinese [27], Persian [28], Spanish [29] and Thai [30], but it has not yet to our knowledge been tested for psychometric properties in a Scandinavian language. Therefore, we chose to translate an existing instrument and test it in a Swedish context.

The aim of the study was to translate the Childbirth Self-efficacy Inventory and to psychometrically test the Swedish version on first- time mothers within the Swedish culture.

## Methods

The present study was carried out in two phases; a translation phase and an evaluating phase of the instrument.

In the first phase, we used forward-backward translation, content and face validity and a pre-test [31,32]. In the second phase, we tested psychometric properties by using a Principal Component Analysis (PCA) and internal consistency which was assessed by Cronbach’s alpha coefficient, inter-item correlations and corrected item total correlations [33].

### Phase 1 translation of the instrument

As a first step, the Childbirth Self-Efficacy Inventory was translated from the original language English, into the target language Swedish. This was performed independently by three midwives who were thus familiar with the area and terminology. The translated versions were then compared and analyzed according to conceptual equivalence, of words and phrases, clarity and discrepancies [31,32]. It was considered of great importance that the terminology used was well-known to the pregnant women and used in Swedish culture. It was concluded that there were only minor discrepancies and these were discussed and refined. Secondly, a back-translation was done into the English language by an independent bilingual professional translator. The translation was blind, meaning that the translator had no prior knowledge of the instrument and had not read the original. The aim of this forward-backward translation is that the instrument would be adjusted to the cultural setting and that the concepts are coherent with the original edition, rather than simple linguistic/literal equivalence [31]. Finally, the back-translated version was compared with the original CBSEI by the authors. No further changes were carried out.

### Face and content validity

The third step in the process was to test the instrument in a panel of experts [32,33]. Two expert panels were used to cover both the delivery area as well as the self-efficacy construct. The instrument was submitted to a panel that consisted of four midwives and one obstetrician working clinically at a delivery ward. The Panel was asked to review CBSEI’s instructions and the items, as well as assessing the appropriateness of the translation concerning comprehension, the words and expressions used and the relevance of the items. During this part they were asked to “think aloud” to verbalize their thoughts. This method provided information on how the CBSEI was cognitively processed, thus clarifying the understanding of the instrument [34]. The first author took notes of what was verbalized and what needed further clarification. An additional expert panel consisting of three researchers in psychology and sports psychology was used to study the instrument with regard to the underlying Self-Efficacy Theory [15]. These members studied the instrument individually and then discussed it together. A written summary of their conclusions was submitted to the authors concluded that the expert panel agreed that the content of the instrument was in line with the self-efficacy theory.

Finally, a fourth step was conducted. According to the recommendations of the World Health Organization [32], it is necessary to pre-test the instrument on the target population. Therefore, the next step was to test the instrument on the group that it was intended for and would be administered to, i.e. pregnant women. In order to assess the comprehension of the translation, each statement and item was read individually and the women were instructed to think aloud, i.e. verbalizing their thoughts while reading the questionnaires. Notes about what was verbalized in the group were taken by the author during the session. The think-aloud method was considered appropriate as it can provide insight into how items are processed and understood [34]. The Swedish version of the CBSEI was read, section by section, and the participants were requested to present comments on the words and sentences used in the questionnaire. They were also told to assess the relevance of the items if whether it was easy to understand or whether something was ambiguous or could be offensive.

### Phase 2: psychometric properties

#### ***Data collection***

The Swedish version of the CBSEI was administrated together with a set of socio-demographic questions. The women completed the forms in the waiting room in connection to a routine antenatal visit.

#### ***Data analysis***

Descriptive statistics were used to describe the participants’ characteristics and preparation for childbirth. The CBSEI consists of four scales, which were analyzed separately, as recommended by Lowe [25]. Descriptive statistics and non-parametric tests (Wilcoxon Signed Ranks Test and Spearman’s rho) were used to describe and compare the scales. Non-parametric tests were used due to skewed distribution on the outcome efficacy scales. The level for statistical significance was set at *p <* 0.05. Furthermore, a principal components analysis (PCA) was performed to explore patterns of relationships between the items [35]. We used an unforced PCA, with a varimax rotation and then an additional analysis with an oblimin direct rotation. The suitability of performing a PCA was assessed with the Kaiser- Meier-Olkin Measure of sampling (KMO) and the Bartlett’s test of sphericity for significance [36,37]. In the analysis of the PCA, we used the following criteria for selecting the components that should be retained. These were: the Kaiser-Guttman criterion with components with an eigenvalue >1, and a visual inspection of Catell’s scree test, looking for the break point where the curve flattens out. Moreover, only components with loadings exceeding > 0.40 were retained [36].

Internal consistency and reliability were analyzed by using an inter-item correlation analysis, a corrected item total correlations test, and Cronbach’s alpha coefficient. A value above 0.3 was considered sufficient for the inter-item and the corrected item total correlations [38,39] and an alpha value that reached 0.70 was considered reliable [38].

The statistical analyses were conducted using IBM SPSS 20.0.

### Participants

In the first phase, the 4th step, of instrument translation, 21 women from four different antenatal education groups at one antenatal clinic were asked to participate. Most of the women were first-time mothers but there were also two women who were pregnant with their second child.

In the second psychometric testing phase, all six antenatal clinics in Halland County, Sweden were enrolled, but one chose to abstain due to renovation. Inclusion criteria for participating in the present study were healthy, pregnant, nulliparous women with a single fetus in cephalic presentation. Furthermore, an additional criterion for participation was that the women could understand the Swedish language sufficiently well to read and fill in the questionnaires.

The women were consequently invited, to participate at 35-42 weeks of gestation when attending their routine antenatal visit. Four hundred and fifty-nine women were asked to participate. Twenty-three women declined participation, most of them due to a lack of time. Out of the 436 women who accepted to participate twenty-six did not return their questionnaires. Thus, 410 women returned their questionnaires. Four of these were excluded ad hoc, because the inclusion criteria did not match. In total 406 cases were assessed to be valid, making a response rate of 93%. The sample size was considered adequate for analyzing psychometric properties using the CBSEI which includes 62 items [38]. All data were collected from January 2011 to June 2012.

### Instrument

The original Childbirth Self-Efficacy Inventory (CBSEI) [25], comprising 62 items, was used in the first instrument translation phase and in the second psychometric phase the Swedish version (Swe-CBSEI) was used.

### Ethical considerations

A written permission was obtained from Nancy K Lowe, who developed the instrument [25]. Furthermore, all participants (i.e. the pregnant women) were informed, both verbally and in written form, about the purpose and the confidentially of the study, and the right to withdraw by their antenatal midwife who had recruited them. A written consent to participate was then returned signed from the participants.

Ethical approval was obtained from the Research Ethics Committee in Lund, Sweden (no.2009/19).

## Results

### Phase 1: translation of the instrument

In the first phase of the study, the translation and back-translation process, the authors reviewed all versions. The objective was to translate the instrument relevance regarding conceptual and cultural equivalence and strive for simplicity. Minor linguistic and stylistic problems were identified in the instructions, these were amended and simplified. For example, in the instructions in part II, a part of the sentence “*when you are pushing your baby out to give birth”* was removed and “*when you give birth*” was retained so that fewer words were used. A common word used in the instrument that was a major language issue concerned the translation of the word “*contractions*”. In Sweden, both “contractions” and the word “pain” are used during childbirth. Sometimes these differed depending on how far the labour process had exceeded (i.e. the word “pain” is more often used during the active phase of labour). We chose to bring this matter to the panel of experts and the parents groups, and these confirmed that the word “pain” was ’the most appropriate. Furthermore, all items were discussed, and some minor changes were made. There were only two items that resulted in a larger discussion and refinement. One of these was item 3 “*using breathing during my contractions*”. This question is not about the usual breathing, but a special breathing technique used during labour, which should be reflected. Therefore, the item was changed to “using *breathing techniques during my contractions”.* The second item discussed was item 9, “*Stay on top of each contraction*”. This item was found to be particularly problematic to translate. This resulted in an equivalent expression “*to follow the rhythm of the contractions”,* which is more common in the Swedish culture. Finally, there were no discrepancies in the backward translation of any words or phrases.

### Content and face validity

The two expert panels reviewed the Swedish version of the CBSEI, and concluded that there were no words that were problematic or culturally inappropriate. They also established the relevance of each item. However, in the clinical panel consisting of midwives and an obstetrician one item was identified as problematic and questioned whether it was relevant, especially for first-time mothers. This item was number 13, “*Think about others in my family*”.

The second expert panel that evaluated the instrument theoretically according to the self-efficacy theory concluded that the instrument had adequate content to measure outcome and self-efficacy expectancy.

Overall, in the first phase of the translation, most of the participating pregnant women had no problems to understanding and completing the Swedish version of the CBSEI. However, some commented that the instructions were repeated, with quite long sentences and that these could be improved upon. Two women raised issues related to the 10-points Likert scale, that this could be refined and reduced to a 5- point scale. Several women pointed out that it was appealing that the statements were positive expressions and there was a consensus that the instrument addressed important aspects of childbirth.

Furthermore, the majority of the items was considered relevant except for one. In all four antenatal groups item 13, “*Think about others in my family*”, was considered problematic. The participants perceived it unclear, whether the item referred to their own family with mother, father and perhaps siblings, or their new family that would be formed with the birth of the baby. If the item referred to their old family (i.e. their own mother, father and siblings), it was considered irrelevant and not applicable to the situation. But we chose not to remove the item in this phase until it had been tested psychometrically.

Finally, there were some first-time pregnant women who felt doubtful as to whether they had been able to answer the CBSEI correctly, as they said in their own words:… *“that they had not had any experiences of childbirth and thereby, no expertise to make judgments*”.

### Phase 2: psychometric properties

#### ***Descriptive findings of the sample***

All 406 nulliparous women aged between 17 and 44 years entered into the study. The mean age of respondents was 28.32 (SD 4.8) and almost everyone was born in Sweden (90.4%) and lived with their partner (94.3%). A majority of the women were working during pregnancy (73.6%) and almost fifty percent (47%) had a college/university education. The women prepared themselves by attending antenatal courses (90.1%). Among the 406 participants, 11 hade internal missing data in a systematic manner, i.e. scales concerning the second stage of labour were scarcely filled out. Missing items were not replaced with any other value, instead we choose to “*exclude cases pairwise”,* an option that allows the variables to be included if they have necessary information.

#### ***Descriptive findings of the Swe-CBSEI***

All four scales of the Swe-CBSEI are cited in this present article as suggested by Lowe [28], that is; Outcome expectancy during the active labour (O -AL), Self-Efficacy expectancy during the active labour (E-AL), Outcome expectancy during the second stage (O-SS) and Self-efficacy expectancy during the second stage (E-SS). Descriptive statistics, regarding mean and standard deviation of the Swe- CBSEI scales apply to the two active labour scales; Outcome-AL, mean 110.9 (SD 25.7). Efficacy-AL, mean 95.0 (SD 23.3). The descriptive statics for the second stage scales were: Outcome-SS, mean 116.2 (SD 27.4) and Efficacy-SS, mean 99.2 (SD 27.5) (Table 1). Histograms of the four scales showed that both of the Outcome expectancy scales were negatively skewed, while the Self-Efficacy expectancy scales were normally distributed, with findings which were consistent with the original study [25]. The descriptive data for each of the scales are presented and compared with Lowe’s [25] findings in Table 1. A Wilcoxon Signed-Ranks Test, revealed a statistically significant difference between the outcome and self-efficacy expectancy, where the outcome expectancy scores were higher for both of the labour stages. The active stage (O-AL and E-AL), *z* = -10.08, *p* < 0.001 and for second stage of labour, (O-SS and E-SS), *z*-11.19, *p* < 0.001). These results indicate that the women made a difference between the behaviour they thought would be useful during labour (outcome expectancies) which they estimated to be higher and the behaviour they believed they were able to carry out (self-efficacy expectancy).

**Table 1:** **Comparisons of the present Swe-CBSEI and Lowe’s (1993) original study for nulliparous women, in terms of descriptive statistics and internal consistency reliability with Cronbach’s coefficient *α***

**Scales**	**Present study, Swe-CBSEI**	**Lowe’s CBSEI (1993)**
	**(n = 406)**	**(n = 264)**
**Outcome-AL**		
Range	30-187	74-150
Mean	110. 9	128.5
Median	116.0	
SD	25.7	14.3
Cronbach’s alpha coefficient *α.*	0.89	0.86
**Efficacy-AL**		
Range	19-150	15-150
Mean	95.0	101.1
Median	95.0	
SD	23.3	21.5
Cronbach’s alpha coefficient *α.*	0.92	0.93
**Outcome-SS**		
Range	20-160	62-160
Mean	116.2	130.0
Median	121.0	
SD	27.4	20.1
Cronbach’s alpha coefficient *α.*	0.94	0.90
**Efficacy-SS**		
Range	16-160	16-169
Mean	99.2	104.4
Median	101.0	
SD	27.5	26.4
Cronbach’s alpha coefficient *α.*	0.94	0.95
**Outcome-Total**		
Range	1-310	156-310
Mean	223.5	258.4
SD	52.7	31.6
Cronbach’s alpha coefficient *α.*	0.95	
**Efficacy-Total**		
Range	35-310	31-310
Mean	191.3	205.5
SD	50.7	45.8
Cronbach’s alpha coefficient *α.*	0.96	

Moreover, a Wilcoxon Signed-Ranks Test was also conducted to compare the differences between the outcome expectancy for the active stage and the second stage (O-AL and O-SS); *z* = -6.26, *p* < 0.001as well as the self-efficacy expectancy for the active stage and the second stage (E-AL and E-SS; *z* = -6.01, *p* < 0.001. These findings were also statistically significant, indicating that the women in the study were able to distinguish between the two labour stages. Finally, the relationship between the outcome expectancy during active labour and the outcome expectancy during the second stage and the efficacy expectancy during active labour and during the second stage of labour was investigated using Spearman’s rho*.* The scales were highly correlated with each other. Findings for the Outcome expectancies for active and second stage of labour was, *r* = .73, n 369, *p* < 0.001 and, *r =* .82, n 373, *p* < 0.001 for the Self-efficacy expectancy for the active stage and the second stage of labour. These findings indicate that one stage (two scales, 31 items) of the inventory could be removed without violating the inventory; it would still measure women’s childbirth self-efficacy during the whole birthing process.

### Construct validity

The Kaiser-Meyer-Olkin scores were non-significant, ranging from 0.925 to 0.938, and Bartlett’s test of sphericity reached statistical significance (p = 0.001) with all four scales. These findings are a fundamental requirement of the PCA and support that the data were suitable for a factor analysis. The Principal Component Analysis with varimax rotation revealed a structure with cross-loadings of twenty-two items (35%). Therefore, an additional analysis with direct oblimin rotation was performed. In this second analysis, three scales (O-AL, O-SS, E-SS) loaded onto a three-component structure, which explained 66.8-71.9% of the total variance. The fourth scale E-AL, loaded onto a two-component structure with a 58.4% explanation of the total variance. All components with an eigenvalue more than >1 showing consistency with the visual Catell’s scree test were selected to be retained as components (Table 2). The underlying concepts of the three-component structure were interpreted by the authors, and labeled self-control, distraction and affirmation in accordance with the essence of the items. We summarize the results from the PCA, with direct oblimin rotation in (Table 3).

**Table 2:** Total variance explained of the Swedish Childbirth Self-Efficacy Inventories subscales

**Subscale**	**Component extracted**	**Eigenvalue**	**Percent of variance**	**Cumulative percent**
Outcome-AL	1	7.77	66.8	51.8
	2	1.23	8.2	60.0
	3	1.01	6. 8	66.8
Efficacy-AL	1	7.36	49.1	49.1
	2	1.40	9.3	58.5
Outcome-SS	1	9.02	56.4	56.4
	2	1.34	8.4	64.8
	3	1.13	7.1	71.9
Efficacy-SS	1	9.09	56.8	56.9
	2	1.29	8.1	65.0
	3	1.00	6.3	71.2

Extraction Method: Principal component Analysis.

Rotation Method: Oblimin with Kaiser Normalization.

**Table 3:** Construct validity of the Swe-CBSEI in terms of patterns matrix for Principal Component Analysis with direct oblimin rotation, component loadings above 0.40

**Items**	**Item no.**	**Scale**	**Component 1**	**Component 2**	**Component 3**
Relax my body	1	O-AL	.845		
	16	E-AL	.879		
	31	O-SS	.818		
	47	E-SS	.904		
Get ready for each contraction	2	O-AL	.711		
	17	E-AL	.818		
	32	O-SS	.767		
	48	E-SS	.758		
Use breathing techniques during labour contractions	3	O-AL	.858		
	18	E-AL	.825		
	33	O-SS	.752		
	49	E-SS	.832		
Keep myself in control	4	O-AL	.541		.457
	19	E-AL	.736		
	34	O-SS	.816		
	50	E-SS	.739		
Think about relaxing	5	O-AL	.774		
	20	E-AL	.822		
	35	O-SS	.818		
	51	E-SS	.804		
Concentrate on an object in the room to distract myself	6	O-AL		.712	
	21	E-AL		.682	
	36	O-SS		.838	
	52	E-SS		.771	
Keep myself calm	7	O-AL	.853		
	22	E-AL	.703		
	37	O-SS	.554		
	53	E-SS	.570		
Concentrate on thinking about the baby	8	O-AL	.668		
	23	E-AL		.526	
	38	O-SS			-.793
	54	E-SS			-.715
Follow the rhythm of the contractions	9	O-AL	.773		
	24	E-AL	.669		
	39	O-SS	.418		-.494
	55	E-SS	.467		-.486
Think positively	10	O-AL	.805		
	25	E-AL	.490	.416	
	40	O-SS			-.678
	56	E-SS			-.615
Not think about the pain	11	O-AL			.770
	26	E-AL		.473	
	41	O-SS	.509	.456	
	57	E-SS	.537	.433	
Tell myself that I can do it	12	O-AL	.786		
	27	E-AL	.462	.457	
	42	O-SS			-.715
	58	E-SS			-.673
Think about others in my family	13	O -AL		.790	
	28	E-AL		.868	
	43	O-SS		.779	
	59	E-SS		.849	
Concentrate on getting through one contraction at a time	14	O-AL	.840		
	29	E-AL	.582		
	44	O-SS	.444		-.521
	60	E-SS	.422		-529
Listen to encouragement from the person helping me	15	O-AL	.772		
	30	E-AL		.507	
	45	O-SS			-.851
	61	E-SS			-.857
Focus on the person helping me in labor	46	O-SS			-.855
	62	E-SS			-.850

The four scales of the Swe-CBSEI; Outcome expectancies for active labour (15 items). Self-Efficacy expectancies for active labour (15 items). Outcome expectancies for second stage labour (16 items). Self-efficacy expectancies for second stage labour (16 items).

### Reliability

Cronbach’s alpha coefficient for the total outcome scale was 0.95 and for the total efficacy scales 0.96. The individual scales varied between 0.92 and 0.94 (Table 2). The values for the corrected item total correlations test were greater than 0.30 for all items except for item 11(Outcome-Active Labour). Furthermore, there were three items on the Outcome Second Stage scale, no. 37, 40 and 44, showing values for the item total correlations test greater than 0.8. Finally, the Inter-item analysis showed a pattern that was recurring. It was the same three questions on the different scales that had the lowest correlations, less than 0.3. These items were; “*Concentrate on an object in the room to distract myself*”, “*Not think about pain*”, *and “Think about others in my family*”*.* These findings indicate that these items are not highly correlated with the other items measuring the same construct and therefore these should be excluded to increase internal consistency.

## Discussion

When an instrument is translated from one language into another, it is fundamental to the quality of translation and thus the validity and reliability that certain steps are being followed [31,32]. We used a forward- backward translation that is considered essential and we also used expert panels in the validation process. The mean scores and standard deviations in the present study were coherent with previous studies [19,25,26,29]. We must bear in mind that in contrast to other studies, both ours as well as Lowe’s [25] study clearly distinguish between nulliparous and multiparous women. It is advisable that the included participants have a similar experience of the subject to be studied, since self-efficacy is influenced by past experience [15,40]. The strength of the present study was the design to utilize inexperienced informants. Of course, we cannot exclude that other experiences may also influence self-efficacy, but the most important factor is at least the same for the participating women. This study confirmed previous findings [20, 26- 28] that women can differentiate between the concepts “outcome efficacy” and “self-efficacy expectations”. The present study also confirmed Lowe’s findings [25] that the women could distinguish between the two labour stages. A major strength of this study is the comparatively large sample size (n = 406). According to Rattray and Jones [37], a sufficient size for an exploratory factor analysis is five responders per item and the CBSEI consists of 62 items. Lowe’s study [25] and the present study are the only ones that have tested the original version with 62 items of the CBSEI and reached the requirements with sufficient numbers of responders according to Rattray and Jone’s recommendations [37]. The Swedish CBSEI revealed a three-component structure, which we named self-control, distraction and affirmations. Drummond & Rickwood [19] questioned the combination of those statements in the same questionnaire and claimed that women do not use both internally and externally focused strategies during labour. But in our earlier findings [14], women expressed that they used both distraction and self-control simultaneously during the early labour process. These previous results from the qualitative study on the latency phase thus support the factor structure in the present study. One may argue that there are different strategies to meet active labour, but the findings of this study support the idea that there is a conceptual coherence between the labour stages. Health care should be based on individual preferences and make efforts accordingly. If self-efficacy is low prior to labour, the psychological well-being of the woman may be affected. Self-efficacy is not a trait, it is considered to be a state [15]. A state can change and is contextual and therefore, it may be influenced. We believe that CBSEI could act as a tool to identify women who need additional support during pregnancy. It may also be used to evaluate antenatal preparations and other interventions during pregnancy. The CBSEI has been translated into a variety of languages [27-30]. However, it is not a tool that has frequently been used in clinical practice in those countries. Although, fairly recently, some researchers have used the instrument in interventions studies to evaluate birth balls [41] and for evaluating educational software [42].

Instruments used clinically require not only that the instrument is valid and reliable but it must also be easy to administrate and easy to respond to. The CBSEI is a rather complex instrument because it consists of four scales with repeated questions related to various parts of labour and childbirth. The repeated questions may seem pointless to the respondents, who may not understand that the questions refer to different parts of labour, bringing about a feeling of being asked the same thing over and over again, ending up in no compliance. There were some women in the current study that had crossed out the questions in the second stage and written “*I have already answered these questions*”. This misunderstanding may derive from different perspectives of labour, where the women see labour as a continuous process and not as clearly defined parts of childbirth. To distinguish between the stages may just be of interest for the professionals [43].

Given the complexity of CBSEI, leads us to propose that the inventory should be reduced when it comes to the number of items and shortened to only address the labour process without differentiating between the stages. This is supported in the present study by the highly correlated scales for both stages. Ip et al. [44] have already developed a shorter Chinese version, CBSEI-C32 and Tanglakmankhong et al. [30] have raised the question. In our study, there were items that had loadings of over 0.4 on more than one component, indicating that they have some dimensions that they share. Additional findings that indicates that the number of items of the instrument could be reduced, is for example the Corrected-item- Total Correlation values. Three values were above 0.8, indicating that these items measure the same as other items on the scale [38,45]. Furthermore, high values of Cronbach’s alpha greater than 0.9 may indicate the same thing [38]. Finally, there were inter-item correlations less than 0.3, indicating that some items do not measure the underlying construct [38,45]. These items were also those witch were questioned whether they were relevant or not by the participants and the expert group. More work is needed to further investigate and scrutinize these questions and perhaps a confirmatory factor analysis can be conducted to strengthen the concept [46].

With increasing rates of cesarean sections world-wide, we should adopt a salutogenic approach, which means that caregivers need to help women identify their strengths and facilitate women’s use of their own strengths. After having testing the instrument for internal a conceptual properties it is of utmost importance to identify women’s strengths and weakness such as sense of coherence, self-esteem and anxiety. It is equality important to find out how high score on CBSEI predicts interventions and labour outcomes during birth.

There are some limitations in this study. The sample was a consecutive sample in one regional part of Sweden. The sample was relatively homogeneous, with very little ethnic diversity, well-educated women and almost everyone was living with their male partner. Therefore, we cannot claim that it is representative for all pregnant women in Sweden.

## Conclusions

The CBSEI was successfully translated and tested by face and content validity. In general, the findings for the Swedish Childbirth Self-efficacy Inventory (Swe-CBSEI) showed a three-component solution with acceptable construct validity and with high internal consistency reliability. The principal component analysis findings suggest that the CBSEI is a reliable and valid instrument that can act as a tool to identify those women who need additional support and to evaluate efforts to improve women’s self-efficacy during pregnancy. Results also indicate that the scales can be shortened, and the scales regarding expectancy and self-efficacy during the second stage can very well be excluded since it does not contribute in any decisive way to the understanding of self-efficacy for the birthing process.

## Competing interests

The authors declare that they have no competing interests.

## Authors’ contributions

All authors contributed to the initiation of the project and contributed to the design (IMC, KZ, EN). The first author (IMC) collected the data and performed the statistical analysis. All three authors interpreted and discussed the results and read and approved the manuscript.

## Authors’ information

The first author, I-MC, is a midwife at Halmstad County Hospital. I-MC is also a doctoral student in in reproductive health, at the department of Women’s and Children’s health at Karolinska Institutet, Stockholm, Sweden and at Halmstad University. The second author, KZ is a registered nurse and associated professor, at the School of Social and Health Sciences at Halmstad University and at Örebro University, Sweden. KZ research focus is on health innovation and on supportive health processes. The third author EN, is a midwife and professor at the department of Women’s and Children’s Health at Karolinska Institutet, Stockholm.

## Pre-publication history

The pre-publication history for this paper can be accessed here:

http://www.biomedcentral.com/1471-2393/14/1/prepub
